# Hemp to limit diffusion of difenoconazole in vegetable garden soils

**DOI:** 10.1016/j.heliyon.2019.e02392

**Published:** 2019-09-03

**Authors:** Clothilde Léchenault-Bergerot, Nadia Morin-Crini, Steffi Rocchi, Eric Lichtfouse, Gilles Chanet, Grégorio Crini

**Affiliations:** a UMR 6249 Chrono-Environnement, Université Bourgogne Franche-Comté, 16 Route de Gray, 25000, Besançon, France; b Parasitologie Mycologie, Centre Hospitalier Régional Universitaire, 25030, Besançon Cedex, France; c Aix Marseille Univ, CNRS, IRD, INRA, Coll France, CEREGE, Aix-en-Provence, France; d Eurochanve, 7 Route de Dijon, Arc-les-Gray, 70100, France

**Keywords:** Environmental science, Materials science, Materials application, Environmental chemistry, Difenoconazole, Hemp, Adsorption, Soil, Pesticide

## Abstract

Triazole molecules are used to manage invasive aspergillosis, a fungal infection mainly due to *Aspergillus fumigatus*. *A. fumigatus* is not a phytopathogen, but, as it is widespread in soils, triazole fungicides have an unintended impact on it, selecting resistant populations’ in environment. Thus, to maintain our ability to control fungal infections, whether in human health or agriculture, reduce the impact of the use of triazoles in the environment is important, notably limiting their diffusion in soils. Here we tested a hemp-based material as adsorbent to limit the spread of difenoconazole, a triazole fungicide, in vegetable soils. We studied the effects of contact time, material dose, difenoconazole concentration, and organic content of the soil using batch mode and percolation methods. Batch experiments showed that the material exhibited high adsorption capacities toward difenoconazole. Removal from the soil water increased from 46.6% using 0.35 g hemp per kg of soil to 77.0% using 1.75 g hemp per kg, for a contact time of 15 min and an initial difenoconazole concentration of 1.2 mg/L. For a contact time of 240 min, the removal was 93.5%. Percolation experiments showed that the quantity of difenoconazole removed was greater than the amount obtained by batch method: 41.9% of removal with only one passes of solution at a concentration of 12 mg/L is obtained through percolation technique whereas, with similar conditions, only 20% of removal is obtained by batch method, i.e. after 1 min of contact. The removal was strongly dependent on the number of passes: the values increased from 57.0% to 91.0% with increasing the number of passes from 1 to 15. Addition of hemp to soils allows to remove efficiently the difenoconazole fungicide from soil water. Hemp-based felt is a new and safe adsorbent that can be applied in agriculture to limit crop contamination.

## 1 Introduction

The agricultural industry uses pesticides to optimize food protection for the growing human population (Berger et al., 2017; Silva et al., 2019). Once crops are infected by fungi, no curative treatment is actually available, leading to crop loss. As a consequence, preventive treatments are often applied. Among treatment options, triazole-based formulations are preferred in agriculture because they specifically target fungi (Price et al., 2015). Triazole molecules are broad-spectrum fungicides used for disease control in cereals, vegetables, fruits and other field crops (Hillocks, 2012; Rocchi et al., 2015). They represent 26.1% of total fungicides sales for agriculture and horticulture in Europe, with 31 molecules available (Fisher et al., 2018).

However, triazole molecules do not only control the targeted pathogenic fungi, such as the species responsible for septoria, *fusarium* ear blight and rust, but have also unintended impacts on other non-phytopathogenic fungi (Avenot et al., 2016). Among them, *Aspergillus fumigatus* is a collateral damage especially worrying. This fungus causes invasive aspergillosis in immunocompromised patients, an invasive fungal infection with a mortality rate (Denning and Bowyer, 2013) of 50% which can reach 100% in cases of infections involving antifungal resistant strains. Medical triazole antifungal drugs are indeed used to treat patients, but many cases of antifungal resistance have been reported for 20 years.

**Scheme 1:**
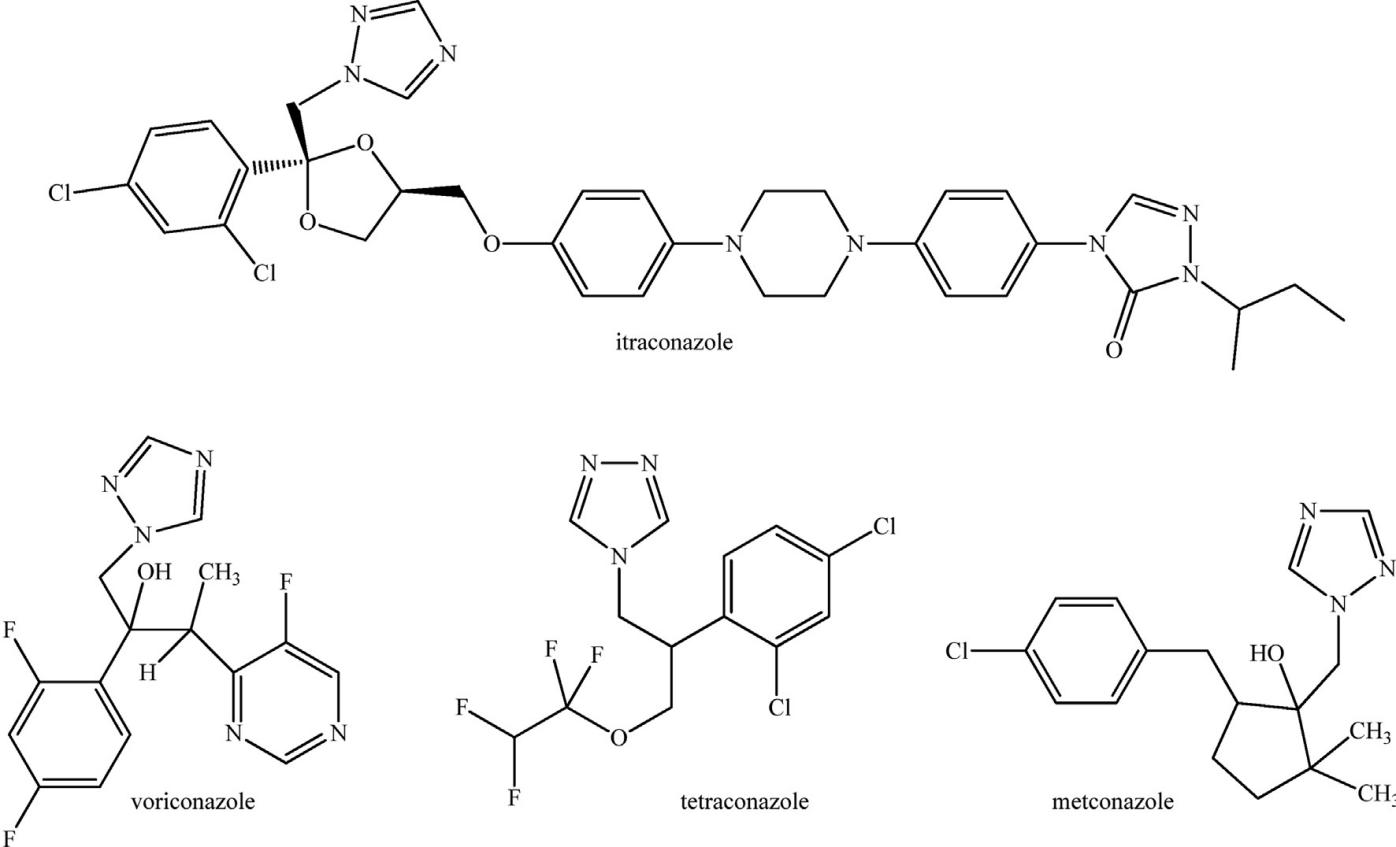
Chemical structures of triazole molecules used in medicine, e.g. voriconazole and itraconazole, and agriculture, e.g. tetraconazole and metconazole.

Two routes of resistance selection are described: the first is linked to the long-term treatment of patients (Camps et al., 2011), the second is likely caused by fungicides largely used in the environment for crop protection (Snelders et al., 2012). Triazole molecules used in medicine and agriculture have similar chemical structures as showed in Scheme 1. Thus, resistant strains with spontaneous mutations are selected by fungicides, or sensitive strains can become resistant through phenotypic plasticity (Hokken et al., 2019). These environmental resistant strains, in contact with fungicides in fields, would be also resistant to antifungal therapies (Verweij et al., 2016). In this context, we recently studied a collection of environmental samples in Eastern France to determinate whether sawmills (Jeanvoine et al., 2017) and market gardens (Rocchi et al., 2018), recognized as huge consumers of triazole molecules, were affected by the presence of azole-resistant *A. fumigatus*. The higher rate of resistant strains was found in market gardens, which mainly used difenoconazole as fungicide, available since 2008 in Europe (Commission Directive, 2008/69/EC). Thus, to reduce resistance, limiting the contact between *A. fumigatus* and triazole molecules clearly appears necessary, while maintaining their efficient action against phytopathogenic molds.

Chemical treatments were recently proposed for the removal of pesticides present in wastewaters (Alonso et al., 2014; Carra et al., 2016; de Aguiar et al., 2019). To our knowledge, studies on their elimination in soils are however rare. An alternative would be to limit the difenoconazole diffusion in soils. For this, we studied the interaction of difenoconazole with a hemp-based material used as adsorbent. Hemp, an annual plant, is an interesting raw material due to its ease of production (no pesticides, rapid growth), low-cost and versatility. Hemp has numerous applications, e.g. textile and paper industries, building and insulation, cosmetics, food, and composites (Bouloc, 2013). However, applications in environmental chemistry are rare. Recently, hemp in fibre or felt forms has been proposed for metal removal from aqueous solutions (Morin-Crini et al., 2018; Pejić et al., 2011; Tofan et al., 2016, 2015; Vukčević et al., 2014).

In this work, we propose for the first time the use of hemp to capture triazole fungicides before reaching soil. Studies concerning the effects of contact time, material dose, and difenoconazole concentration were evaluated using two analytical methods, i.e. batch method and percolation technique. The effect of organic content of soils was also investigated in order to confirm our previous hypothesis that more resistant strains in soils could be related with huge rate of organic matter (Rocchi et al., 2018). This could be explained by the stronger selection pressure exerted by fungicides more retained in soils containing high levels of organic matter (Čadková et al., 2013; Komárek et al., 2010).

## 2 Materials and methods

### 2.1 Materials

**Scheme 2:**
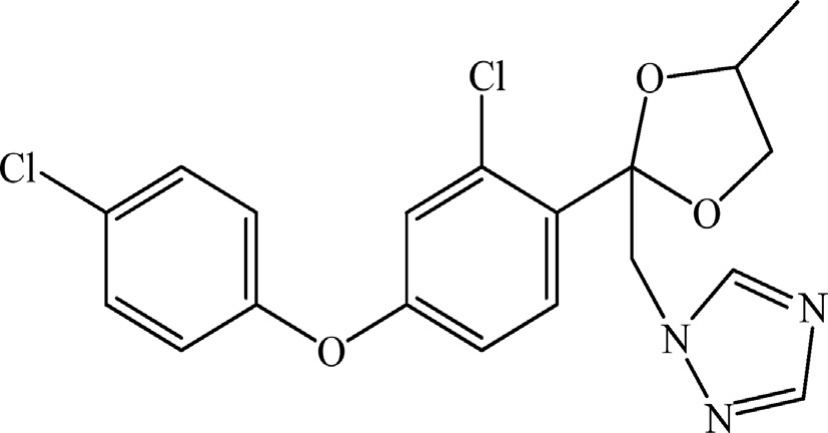
Chemical structure of difenoconazole or 1-[[2-[2-chloro-4-(4-chlorophenoxy)phenyl]-4-methyl-1,3-dioxolan-2-yl]methyl]-1,2,4-triazole.

Difenoconazole is the common name for 1-[[2-[2-chloro-4-(4-chlorophenoxy)phenyl]-4-methyl-1,3-dioxolan-2-yl]methyl]-1,2,4-triazole in the IUPAC nomenclature. Difenoconazole, Pestanal®, CAS number 119445-68-3, analytical standard, was purchased from Sigma Aldrich (Saint Quentin Fallavier, France) and used without purification. Chemical structure is depicted in Scheme 2. Other characteristics are: molecular formula C_19_H_17_Cl_2_N_3_O_3_, molecular weight 406.2 g/mol, solubility in water 15 mg/L at 20 °C, pK_a_ 1.07, and Log K_ow_ 4.36 at 25 °C.

Acetonitrile Distol®, a range of products for organic trace analysis, was obtained from Fisher Scientific (Illkirch, France, www.fishersci.fr). Sodium chloride (purity: 99.5%) was purchased from Sigma Aldrich (Saint Quentin Fallavier, France). Versylene® Fresenius sterile water used for the aqueous synthetic solutions was obtained from Fresenius Kabi (Sèvres, France).

The hemp-based material in felt form was obtained from Eurochanvre, Arc-lès-Gray, France. The hemp felt was made of 100% fiber (75% cellulose, 15% hemicellulose, 3% lignin and 5% pectin) and its thickness is about 5 mm. Three diameters of hemp disk were used: 2.5 cm (Hemp 1), 3.5 cm (Hemp 2), and 4.5 cm (Hemp 5). Their masses were respectively: 0.35 g, 0.7 g, and 1.75 g.

Three autoclaved soils were used: 1) SOIL9.5: a soil containing 9.5% of organic matter (pH = 5.96) collected in an organic market garden in the *Bourgogne Franche-Comté* region (France), sifted at 5 mm, oven-dried, crushed with a pestle, and again sifted at 1 mm; 2) SOIL3.5: a soil composite containing 3.5% of organic matter (pH = 6.18) made by mixing 30% (w/w) of the SOIL9.5 sample with Fontainebleau® sand (Buchi, Rungis, France); and 3) SOIL0: Fontainebleau® sand, which does not contain organic matter. The characteristics of the clay silt organic SOIL9.5, determined by an accredited French laboratory (SADEF, Aspach, France) were: 9.5% of organic matter (NF ISO 14235), pH = 5.96 (NF ISO 10390); fractions of clay particles (<2 mm) 35.8 %, silt particles (2–50 mm) 56.9 %, sand particles (50–2000 mm) 7.2 % according to the NFX 31–107 French norm; total N content 3.77 g kg^−1^ (NF ISO 13878); total P content 0.24 g kg^−1^ (NF X 31–160); cation exchange capacity (CEC) 23.9 cmol kg^−1^ (NF X 31–130).

### 2.2 Sample preparation

Aqueous synthetic solutions of difenoconazole dissolved in a minimal quantity of acetonitrile (1% v/v) were prepared at two concentrations: C1 = 1.2 mg/L (equivalent to the concentration used in market gardens – solution S1) and C2 = 12 mg/L (equivalent to the concentration used in market gardens multiplied by ten – solution S12). Initial solution pH was 5.9 ± 0.1. The two aqueous solutions were prepared twice and analyzed. The experimental errors were 0.33% and 0.19% for C1 and C2, respectively.

### 2.3 Experimental procedures

The experiments were conducted using two adsorption-oriented methods: a batch method and a percolation technique. The first set of experiments was realized using a batch method detailed in previous works (Crini et al., 2017; Morin-Crini et al., 2017), for which kinetic and adsorption capacities of hemp were determined. For kinetic experiments, a hemp disk (0.35 g) was added to 50 mL of the solution 12 in a tightly closed glass flask and stirred on a thermostatic mechanical shaker operating at 250 rpm for various times, ranging from 5 to 240 min. The experiments were conducted at 25 ± 1 °C without changing the initial pH of the solution. For adsorption experiments, the effect of difenoconazole concentration and of disk mass was tested. Thus, 3 masses of hemp disk, i.e. 0.35 g (Hemp 1), 0.70 g (Hemp 2) and 1.75 g (Hemp 5), were added to 50 mL of solution S1 and solution S12 and stirred on a thermostatic mechanical shaker operating at 250 rpm during 15 min. Our objective was to demonstrate that the percentage of difenoconazole removal increased with the hemp dose, even for a low contact time (15 min is more realistic). The removal of difenoconazole was expressed in percentage of abatement, representing the ratio between the amount of adsorbed difenoconazole and its initial amount. Experiments were performed in triplicate. The repeatability has been validated (relative standard deviation below 20%).

**Scheme 3:**
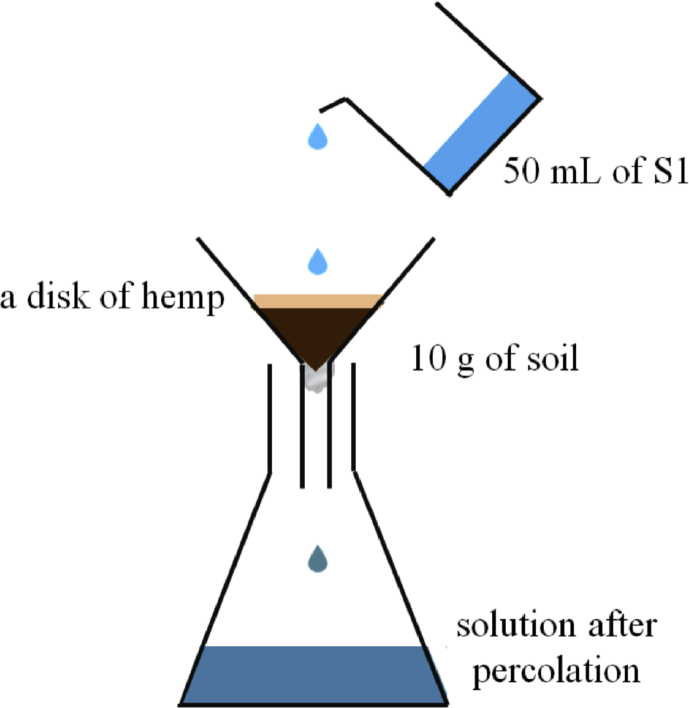
Percolation procedure: this is an extraction process that involves the slow descent of solution S1 through the soil, in absence or presence of a hemp disk; the number of passes through the system was 1, 5, 10 or 15.

For the percolation procedure, the system illustrated in Scheme 3 has been realized thanks to a funnel put on a flask to percolate solution 12 through the hemp. This technique is similar to an open column method. First, 50 mL of solution 12 was poured in the funnel onto 2 masses of hemp disk (0.350 g, 0.700 g). The flow-through was collected after 1, 10 or 15 passes on the same hemp disk. Secondly, the same experiments were performed in presence of soils. 50 mL of solution 12 was then poured on the 3 types of soils (1 and 5 passes) with or without hemp disk (0.700g). The hemp material is placed above 10 g of soil and a piece of cotton was added at the bottom of the funnel (Scheme 3). The removal of difenoconazole was also expressed in percentage of abatement/removal. To correct any adsorption of difenoconazole on container and cotton, control experiments were also carried out in the same conditions. Experiments were performed in triplicate.

The pH of all solutions was measured before and after experiments. It was noted during the experiments a slight pH variation did occur at the end of each experiment, i.e. an increase of between 0.2 and 0.3.

### 2.4 Sample extraction and analysis

The analytical methodology for the liquid-liquid extraction and quantification of difenoconazole is based on a method recently developed by our group (Morin-Crini et al., 2017). Ten milliliters of the solution after adsorption were added to 20 mL of acetonitrile and 3 g of NaCl and stirred by a magnetic plate for 10 min. The supernatant organic layers were recovered using a separating funnel and adjusted to 20 mL with acetonitrile. Then sample extracts were analyzed on a system composed of gas chromatography (GC) apparatus and a triple quadrupole spectrometer (GC-MS/MS, Agilent, Massy, France). The GC-MS/MS optimized parameters of the triazole studied are: precursor ion 265, product ion 202 and 139, collision energy 36 and 40 V, retention time 34.366 and 34.485 min, and limit of detection 0.28 μg/L.

Soils of the percolation procedure have also been extracted for analysis. Ten grams of soil were added to 5 mL of water, 20 mL of acetonitrile and 3 g of NaCl, stirred by a vortex for 3 min. The supernatant organic layers were recovered using centrifugation at 3500 x g for 5 min and adjusted to 20 mL with acetonitrile. Sample extracts were then analyzed as previously described (Morin-Crini et al., 2017). The portion of difenoconazole retained by hemp has been deducted by subtracting the concentration found in the soil from the concentration found in the flow through.

## 3 Results and discussion

### 3.1 Effect of contact time

**Fig. 1:**
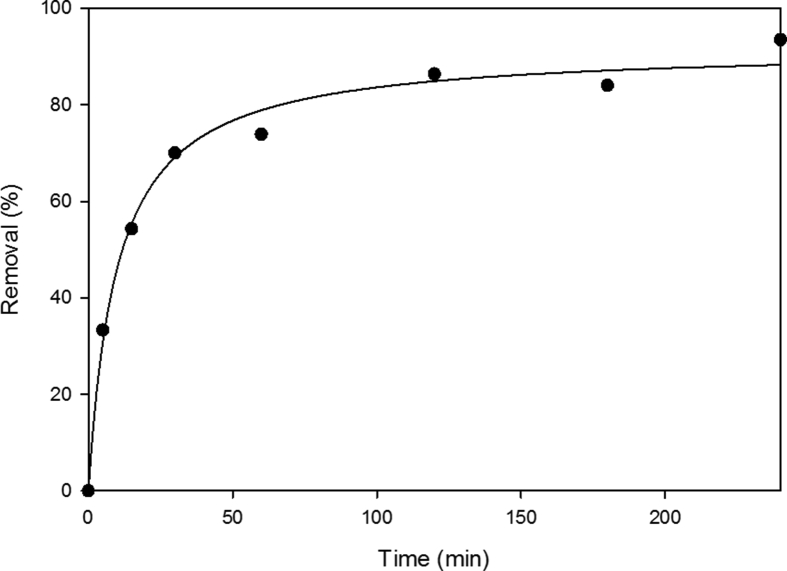
Effect of contact time on difenoconazole adsorption (in %) using hemp (conditions: volume 50 mL; solution at 12 mg/L; hemp dose = 0.35 g; agitation speed = 250 rpm; temperature = 25 °C; n = 3). In these batch experiments, the amount of difenoconazole adsorbed was expressed in percentage removal.

In order to optimize the design of an adsorption system to remove fungicide from solutions, it is important to establish the most appropriate contact time used in batch experiments. Fig. 1 shows the amount of difenoconazole adsorbed by a hemp-based material versus the contact time for concentration of difenoconazole of 12 mg/L (solution S12). The amount of fungicide adsorbed increased with contact time until reaching a constant value where no more fungicide was removed from the solution. These kinetic results indicated that adsorption process was uniform with time and can be considered very fast because of the largest amount of difenoconazole adsorbed to the material within the first 60 min. The process could be divided in three regimes: the removal is increased instantly at initial stages, from 5 to 60 min, e.g. after only 5 min, 33.3% of fungicide was removed; then the removal keeps increasing gradually from 60 to 120 min, until the equilibrium is reached and remains constant. For a contact time of 240 min, the removal was 93.5%, indicating strong interactions between difenoconazole and binding sites present in the main fiber constituents, i. e cellulose, hemicellulose and lignin. The remaining concentration of difenoconazole become asymptotic to the time axis after 90 min of shaking and the amount of difenoconazole showed no significant difference when the contact times were longer than this. Similar results were obtained with a concentration of 1.2 mg/L.

### 3.2 Effect of hemp dose

Adsorbent dose is an important parameter in adsorption phenomena, influencing the number of adsorption sites. The adsorption of difenoconazole on hemp was studied by changing the quantity of hemp in the solution, i.e. 0.35 g (Hemp 1), 0.7g (Hemp 2) and 1.75 g (Hemp 5), whilst maintaining contact time (15 min), agitation speed, and temperature constant. The experiments were conducted at two difenoconazole concentrations, i.e. 1.2 mg/L (solution S1) and 12 mg/L (solution S12). We chose a contact time of 15 min to show differences in fungicide elimination. Indeed, we performed the same experiments with the three doses at a contact time of 120 min and found that all difenoconazole was eliminated.

**Fig. 2:**
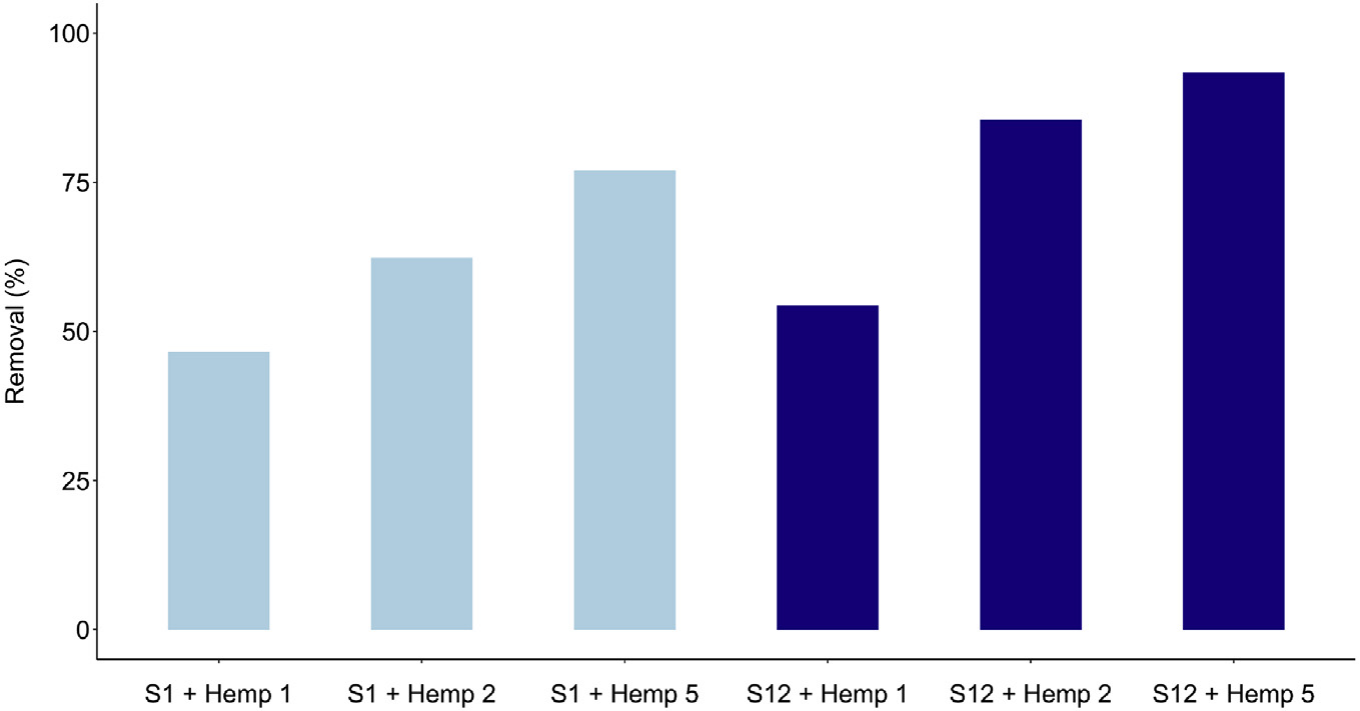
Effect of hemp dose on difenoconazole removal (in %) at two fungicide concentrations, 1.2 mg/L for solution S1 and 12 mg/L for solution S12 (conditions: batch experiments; 0.35, 0.7 and 1.75 g for Hemp 1, Hemp 2 and Hemp 5, respectively; volume 50 mL; contact time = 15 min; agitation speed = 250 rpm; temperature = 25 °C; n = 3).

The results reported in Fig. 2 showed that the percentage of difenoconazole removal increased with the hemp dose. The amount of difenoconazole per unit mass of material, expressed in % removal and at a contact time of 15 min, increased from 46.6% to 77% for solution S1 with increasing dose from Hemp 1 (0.35 g) to Hemp 5 (1.75 g, this mass being 5-fold higher). These results demonstrated that, even for a low contact time, the removal obtained by hemp is significant and reproducible (n = 3). Thus, hemp in felt form is an efficient material for fungicide removal. At higher concentration (solution S12), the removal increased from 54.3% to 93.4% with increasing hemp dose from 0.35 g to 1.75 g. As expected, the removal is dependent on concentration. To obtain better results at higher concentrations, it is necessary either to increase the contact time or to increase the mass of hemp.

### 3.3 Column setup

**Fig. 3:**
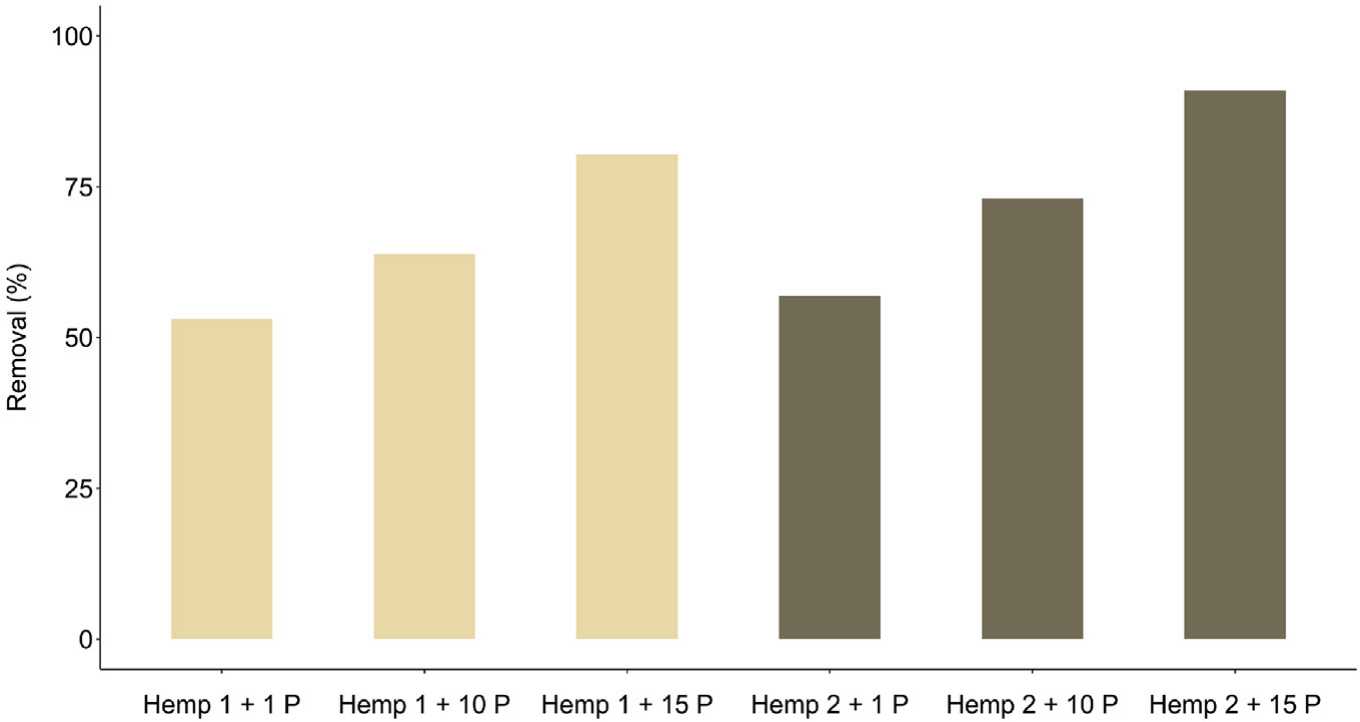
Effect of the number of passes (P) of the fungicide solution in the column on the difenoconazole removal (in %) at two doses of hemp material (conditions: percolation experiments; Hemp 1 = 0.35 g, Hemp 2 = 0.7 g; difenoconazole concentration = 12 mg/L, temperature = 25 °C; n = 3).

In a second experiment, we used a percolation method, similar to an open column (Scheme 3). Two hemp doses were used, i.e. 0.35 (Hemp 1) g and 0.70 g (Hemp 2) and the initial difenoconazole concentration was fixed at 12 mg/L. The removal increased with increasing amount of hemp material as reported in Fig. 3. It was also observed that the removal was strongly dependent on the number of passes. The removal increased from 53.1% to 80.4% and from 57.0% to 91.0% for Hemp 1 and Hemp 2, respectively, with increasing the number of passes from 1 to 15.

### 3.4 Effect of organic matter content

**Fig. 4:**
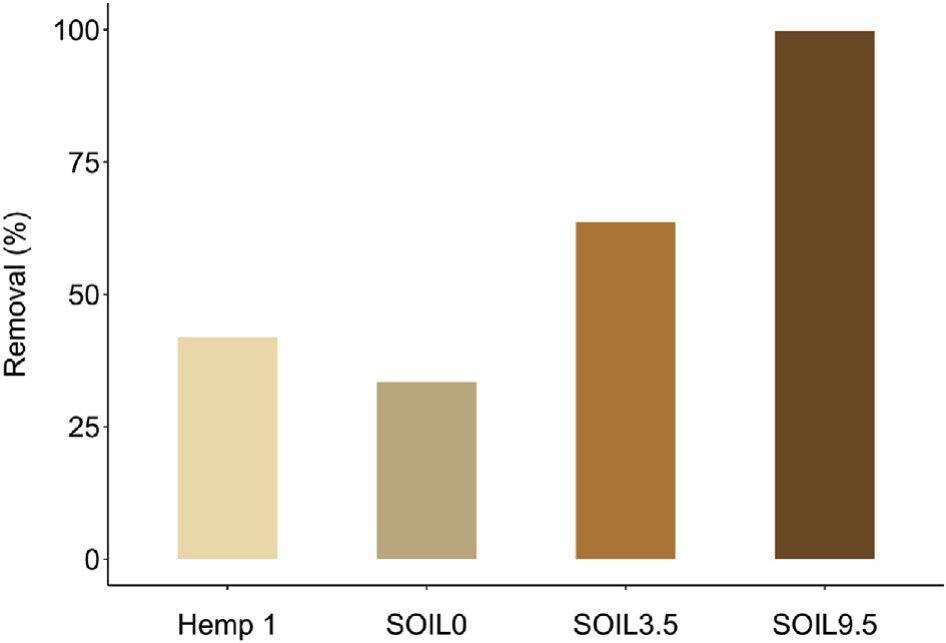
Comparison of difenoconazole removal (in %) using hemp and three soils at a fixed difenoconazole concentration of 12 mg/L: Hemp 1 (mass: 0.35g), SOIL0 = Fontainebleau® sand, SOIL3.5 and SOIL9.5 = soils with 3.5% and 9.5% of organic matter, respectively (conditions: percolation experiments; number of passes = 1; temperature = 25 °C; n = 3).

Fig. 4 compares the difenoconazole removal of various soils having different organic matter content: SOIL0 which does not contain organic matter (Fontainebleau® sand) and two soils containing 3.5% (SOIL3.5) and 9.5% (SOIL9.5) of organic matter. The removals were 99.8%, 63.7% and 33.4% for SOIL9.5, SOIL3.5 and SOIL0, respectively. The results showed that they were dependent on organic matter content. This observation was consistent with the literature which reported a poor rate of organic matter retained less triazole fungicides because of their hydrophobicity (Komárek et al., 2010). For hemp material, the quantity of difenoconazole removed was greater than the amount obtained by the batch method: 41.9% of removal with only one passes of solution at a concentration of 12 mg/L is obtained through percolation technique whereas, with similar conditions (hemp dose, fungicide concentration) only 20% of removal is obtained by batch method, i.e. after 1 min of contact.

### 3.5 Effect of hemp to limit difenoconazole diffusion

**Fig. 5:**
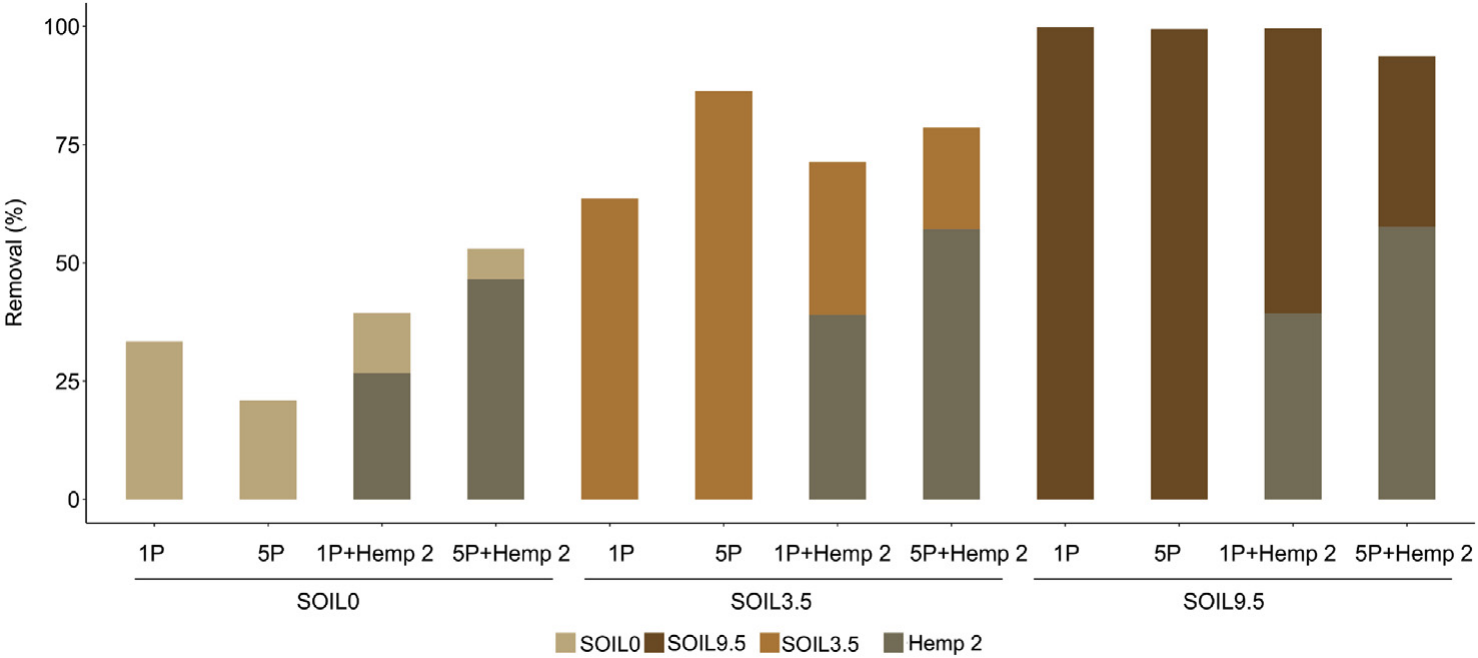
Effect of the number of passes on difenoconazole removal (in %) using various soils in absence or presence of hemp (conditions: percolation experiments; materials: Hemp 2, SOIL0, SOIL3.5 and SOIL9.5; solution at 12 mg/L; P = number of passes; temperature = 25 °C).

Fig. 5 shows the effect of the absence or presence of hemp on difenoconazole removal using three soils. The experiments were conducted at 1 and 5 passes of solution S12 (concentration: 12 mg/L). After 1 or 5 passes, in absence of hemp, the order of abatement was the following: SOIL9.5 > SOIL3.5 > SOIL0. For SOIL9.5, all the difenoconazole concentration present in solution was adsorbed by the soil. For SOIL0 (Fontainebleau® sand), after 5 passes, the removal decreased due to the possible desorption of adsorbed molecules. In presence of hemp, global abatements were systematically better and are in agreement with those obtained by batch method. The hemp retained more after 5 passes of the solution. Hemp can retain more than 50% of difenoconazole after 5 passes of solution, and this result was independent on organic matter.

## 4 Conclusions

Traditionally, hemp-based products in the form of mulch or felt, supplied mainly in rolls, are used for horticultural mulching. For example, these materials are a biodegradable layer of fibers used to create a suitable planting environment that warms the soil, retains moisture, and deters weeds. The concept we are working on is to use hemp in vegetable gardens and small crops (e. g. vines) and sawmills, in order to limit the spread of pesticides, once sprayed, to the ground. In sawmills, hemp felt could be placed under soaking basins and drying areas to recover excess treatment before release. In small crops and vegetable gardens, hemp could be spread around the seedlings and replaced regularly. After use, the felts could then be used as fuel.

In this study, our results demonstrated that hemp-based felt was an efficient material for the removal of difenoconazole present in aqueous solutions. In batch mode, hemp was able to remove difenoconazole in 60 min. More interesting results were obtained using a percolation technique. Hemp could be able to decrease difenoconazole concentration in soils after use and thus limit the contact with the fungal pathogens. Difenoconazole adsorption depended also on the organic content of the soil. As agricultural textiles, hemp-based felts can provide an ecological protection against pesticides to prevent their spread in the soil. The observation of a decrease in triazole concentration using hemp confirms our hypothesis that the different adsorption rate of triazole molecules in soils could be a factor influencing the selection of resistant strains of *A. fumigatus*. Given that a few studies already exist on triazole sorption on soils (Čadková et al., 2013), further studies on other soils will be investigated.

## Declarations

### Author contribution statement

Clothilde Léchenault-Bergerot, Steffi Rocchi: Performed the experiments; Contributed reagents, materials, analysis tools or data.

Nadia Morin-Crini: Conceived and designed the experiments; Analyzed and interpreted the data; Wrote the paper.

Eric Lichtfouse, Gilles Chanet: Contributed reagents, materials, analysis tools or data.

Grégorio Crini: Analyzed and interpreted the data.

### Funding statement

This work was supported by the FEDER, Fonds Européen de Dévelopement Régional (project: Innovative materials for wastewater treatment).

### Competing interest statement

The authors declare no conflict of interest.

### Additional information

No additional information is available for this paper.
